# Determinants of Full Breastfeeding at 6 Months and Any Breastfeeding at 12 and 24 Months among Women in Sydney: Findings from the HSHK Birth Cohort Study

**DOI:** 10.3390/ijerph17155384

**Published:** 2020-07-27

**Authors:** Ritesh Chimoriya, Jane Anne Scott, James Rufus John, Sameer Bhole, Andrew Hayen, Gregory S. Kolt, Amit Arora

**Affiliations:** 1 School of Health Sciences, Western Sydney University, Penrith 2751, NSW, Australia; r.chimoriya@westernsydney.edu.au (R.C.); g.kolt@westernsydney.edu.au (G.S.K.); 2 School of Public Health, Curtin University, Perth 6845, WA, Australia; jane.scott@curtin.edu.au; 3 Translational Health Research Institute, Western Sydney University, Locked Bag 1797, Penrith 2751, NSW, Australia; rufus.benaud11@gmail.com; 4 Rozetta Institute, Sydney, NSW 2000, Australia; 5 Oral Health Services, Sydney Local Health District and Sydney Dental Hospital, NSW Health, Surry Hills 2010, NSW, Australia; sameer.bhole@health.nsw.gov.au; 6 Sydney Dental School, Faculty of Medicine and Health, The University of Sydney, Surry Hills 2010, NSW, Australia; 7 Australian Centre for Public and Population Health Research, Faculty of Health, University of Technology Sydney, Ultimo 2007, NSW, Australia; andrew.hayen@uts.edu.au; 8 Discipline of Child and Adolescent Health, Sydney Medical School, Faculty of Medicine and Health, The University of Sydney, Westmead 2145, NSW, Australia

**Keywords:** breastfeeding, deprivation, Australia, infant, low socioeconomic status

## Abstract

The aim of this study was to report on breastfeeding duration up to 24 months and determine the predictors of breastfeeding duration among women in South Western Sydney, one of the most culturally diverse and socioeconomically disadvantaged regions of New South Wales (NSW), Australia. Mother–infant dyads (*n* = 1035) were recruited to the Healthy Smiles Healthy Kids birth cohort study. Study data were collected through telephone interviews at 2, 4, 8, 12, and 24 months postpartum. Cox proportional hazards models were used to determine factors associated with the risk of stopping full breastfeeding at six months and any breastfeeding at 12 and 24 months. The majority of mothers (92.3%) had initiated breastfeeding. At six months, 13.5% of infants were fully breastfed, while 49.9% received some breast milk. Only 25.5% and 2.9% of infants received some breast milk at 12 and 24 months, respectively. Lower maternal education level, lower socioeconomic status, full-time employment, maternal smoking during pregnancy, and caesarean delivery were associated with increased risk of stopping full breastfeeding at six months and any breastfeeding at 12 and 24 months. Older maternal age and partner’s preference for breastfeeding were associated with an increased likelihood of continuing any breastfeeding at 12 and 24 months. These findings present a number of opportunities for prolonging breastfeeding duration in disadvantaged communities in NSW.

## 1. Introduction

Breastfeeding is recognised as a public-health priority worldwide. The World Health Organization recommends that infants be exclusively breastfed for the first six months, followed by introduction of nutritious and safe complementary foods whilst breastfeeding is continued for up to two years of age or beyond [1]. On the other hand, the Australian Dietary Guidelines [2] and Infant Feeding Guidelines [3] developed by the National Health and Medical Research Council recommend exclusive breastfeeding for infants until around six months of age, followed by the introduction of solid foods whilst continuing breastfeeding until 12 months and beyond. These recommendations are supported by a large body of evidence underlining the short-term and long-term advantages of breastfeeding for both infants and mothers [4,5].

While there is a consensus on the importance of breastfeeding over the first year of an infant’s life, research suggests that breastfeeding continues to influence the child’s health and development beyond 12 months [5,6]. Besides promoting healthy growth in infants through a nutritionally balanced diet, breast milk confers other health benefits to both children and mothers [4,5,7,8,9,10,11]. Breast milk contains several biologically active substances and components associated with the immune system that result in efficient utilisation of the nutrients and provide active and passive protection against diseases until the second year of a child’s life and beyond [4,5]. Short-term and long-term infant health outcomes include cognitive and neurodevelopment advantages and decreased risk of gastrointestinal and respiratory infections, diabetes, obesity, asthma, and cardiovascular disease [4,5,7,11]. Postpartum and long-term maternal health benefits include increased weight loss, reduced blood loss and stress, and lower risk of type 2 diabetes, hypertension, cardiovascular disease, and breast and ovarian cancers [5,8,9,10].

Early cessation of exclusive breastfeeding has become a global phenomenon, with only 38% of infants globally being exclusively breastfed until four months of life [5]. Whilst the majority of Australian women initiate breastfeeding, breastfeeding duration is shorter than recommended. The 2010 Australian National Infant Feeding Survey (NIFS) [12] reported that over 90% of Australian women had initiated breastfeeding, but only around 60% continued to breastfeed for six months. Similarly, around 42% of infants in the age group 7–12 months were still receiving some breast milk, and only about 7% of the 10–24-month-old infants were receiving breast milk [12]. It is therefore important to continuously monitor breastfeeding rates to identify if the national breastfeeding goals are being met, determine the factors that influence breastfeeding practices, and identify women who are least likely to continue breastfeeding up to the recommended duration and for whom breastfeeding promotion interventions can be targeted [13].

Breastfeeding is intrinsically influenced by a diverse range of medical, socioeconomic, cultural, and psychological factors that differ across populations, regions, cultures, and countries [14,15,16,17]. Undertaking research on breastfeeding practices for specific population subgroups assists in identifying various factors that limit mothers’ ability to continue breastfeeding in line with breastfeeding guidelines. South Western Sydney is a metropolitan area with an estimated population of 966,450, accounting for about 12.5% of the New South Wales (NSW) population [18]. Its resident population is ethnically diverse [18,19], and it is also one of the most socioeconomically disadvantaged regions in NSW [19], with limited data on breastfeeding practices [20,21,22,23]. Moreover, the prevalence and predictors of breastfeeding duration over the first two years of infants’ lives have not been extensively investigated longitudinally in this region.

Low socioeconomic status is one of the major barriers to breastfeeding which contributes to increasing health inequalities in Australian children [24]. As compared to children who are breastfed for a shorter duration, those who are breastfed for a longer duration have lower morbidity and mortality and higher intelligence, and these inequalities may persist throughout their lives [5]. Hence, especially in marginalised population subgroups, addressing health disparities and socioeconomic disadvantage is fundamental to promoting breastfeeding. Based on the socioeconomic disadvantage, it is vital to identify subgroups of mothers at risk of discontinuing breastfeeding and implement strategies to promote breastfeeding as per national and international recommendations. Therefore, this study aims to report the prevalence of full breastfeeding at six months and any breastfeeding at 12 and 24 months, and to determine the predictors of breastfeeding duration among women in South Western Sydney.

## 2. Methods 

### 2.1. Setting and Recruitment

This study analysed data obtained from the Healthy Smiles Healthy Kids (HSHK) study, an ongoing multi-centre birth cohort study in South Western Sydney [25]. The details of the HSHK study have been described elsewhere [20,25]. In brief, this ongoing study includes women from disadvantaged communities who delivered a live infant without severe health conditions, in public hospitals from the former Sydney South West Area Health Service (currently the Sydney Local Health District and the South Western Sydney Local Health District) in 2010. Purposive sampling was used as a procedure to identify hospitals with the intent to recruit the underrepresented socioeconomically disadvantaged population predominant in South Western Sydney [25]. Child and Family Health Nurses recruited mother–infant dyads (*n* = 1035) at their first postnatal home visit at 4 to 6 weeks, and written informed consent was acquired for participation in the study at this time.

### 2.2. Data Collection

Study data on family and infant characteristics were collected through telephone interviews at 2 months and subsequently at 4, 8, 12, and 24 months. Information was collected on family and infant characteristics associated with breastfeeding duration. This study used structured questionnaires adapted from the first and second Perth Infant Feeding Studies [13,26].

### 2.3. Infant Feeding Assessments

This study used breastfeeding definitions provided by the World Health Organization [1]. “Exclusive breastfeeding” is defined as the practice in which an infant receives only breast milk, including milk expressed or from a wet nurse, for six months, with no other complementary (solid) foods or liquids, except for oral rehydration solutions (ORS), drops, and syrups (vitamins, minerals, and medicines). “Predominant breastfeeding” refers to the practice where an infant receives breast milk as the principal source of nourishment, without any complementary foods or liquids (formula and non-human milk), except for water, water-based drinks, fruit juice, ritual fluids, ORS, drops, or syrups. “Full breastfeeding” is defined as the practice in which an infant is either exclusively or predominantly breastfed, whereas, “any breastfeeding” is defined as the practice in which an infant receives breast milk, with or without complementary foods or liquids (formula, fluids, and other milk) [27].

The durations of full and any breastfeeding were determined on the basis of data collected on infant feeding practices over the first 24 months of an infant’s life. This includes the age, in weeks, at which breastfeeding was initiated and stopped, and the age at which water (tap and mineral water), other types of milk (cow milk, flavoured milk, and other milk), liquids (juice, soft drink, cordial, sports drink, iced tea or coffee, powdered drink, flavoured mineral water, and honey), and solid food (core and non-core) were introduced. To calculate the age in months, ages reported in weeks were divided by 4.33 and then rounded; hence, 26, 52, and 104 weeks represent 6, 12, and 24 months, respectively.

### 2.4. Outcome Variables

The outcome variables for this study were full breastfeeding at 6 months, and any breastfeeding at 12 and 24 months.

### 2.5. Explanatory Variables

Several family and infant characteristics known to be related to breastfeeding duration were examined. “Maternal age” was recorded as a continuous variable. “Mother’s occupation” was determined on the basis of census occupation codes (home duties, unskilled, sales/clerical, managerial, and professionals) [28]. The Index of Relative Socioeconomic Advantage and Disadvantage (IRSAD), an index for relative socioeconomic disadvantage established by the ABS, was utilised to define the socioeconomic status [19]. The IRSAD summarises variables that indicate either relative advantage or disadvantage and ranks areas on a continuum, from most disadvantaged to least disadvantaged. Mothers reported their residential postcode, and their socioeconomic status was categorised into Deciles 1 to 10, with Decile 1 representing most disadvantaged and Decile 10 representing the least disadvantaged [19]. “Infant birthweight” was documented as a continuous variable and further classified as ≥2500 g and <2500 g. “Parity” was recorded as a continuous variable and then classified as “primiparous” and “multiparous”. “Mother’s smoking status” and “mother’s alcohol consumption status” during pregnancy were documented as a “yes” or “no” response.

### 2.6. Statistical Analysis

The Statistical Package for Social Sciences, Version 25 (SPSS for MacOS, SPSS Inc., Chicago, IL, USA), was used for data analysis. Baseline characteristics are presented as frequency counts with percentages for all categorical variables. Survival analysis was used to determine breastfeeding duration as it provides a good understanding of breastfeeding practices over time. This approach was used due to the presence of censored data. In our study, “censored data” refers to the data from those cases where there was no discontinuation of breastfeeding either by the end of the study period or by the time the participant dropped out of the study. Cox proportional hazards models were used to identify the predictors associated with the risk of stopping full and any breastfeeding. This model allows joint estimation of the effects of independent variables on the “hazard”, which is the risk of cessation of breastfeeding and can be used to analyse data that contain censored observations.

In the univariable model, the effect of each independent variable on breastfeeding duration was evaluated by using the Kaplan–Meier estimate of survival, and the log-rank test was used to assess the quality of survival curves. Variables with *p* < 0.2 were fitted in the multivariable model. The full model was reduced through backward stepwise regression, while simultaneously assessing the model fitness, in order to prevent dropping non-significant variables which may affect the model fitness. The final model consists of variables, which, when eliminated, cause a prominent deviance change (*p* < 0.05), as compared to the corresponding *X*^2^ test statistic on the relevant degrees of freedom.

## 3. Results

Of the 1500 mothers approached to participate in the HSHK study, 1035 mothers provided written consent (69% response rate). In order to determine the representativeness of the sample size, three short questions on sociodemographic status and chosen method of feeding were also gathered from the non-participant mothers (*n* = 465). There were no significant differences between the mothers who consented to take part in the study and those who did not in terms of maternal age (*X*^2^ = 4.75, *p* = 0.153), educational level (*X*^2^ = 6.65, *p* = 0.328), and infant feeding method (*X*^2^ = 2.46, *p* = 0.813). Overall, 101 mothers either declined to progress with the study or could not be reached after seven attempted telephone calls prior to completing the baseline interview. A total of 934 mothers completed the interviews at two, four, and eight months (90.24% retention rate). The total sample dropped to 900 mother-infant dyads by the interview at 12 months (86.96% retention rate) and 795 by the interview at 24 months (76.81% retention rate). There were no differences in the age, education level, and infant feeding method of those who completed the interviews at 12 and 24 months and those who withdrew from the study (data not reported). Table 1 outlines the study population’s family and infant characteristics, along with breastfeeding duration up to 24 months.

**Figure 1:**
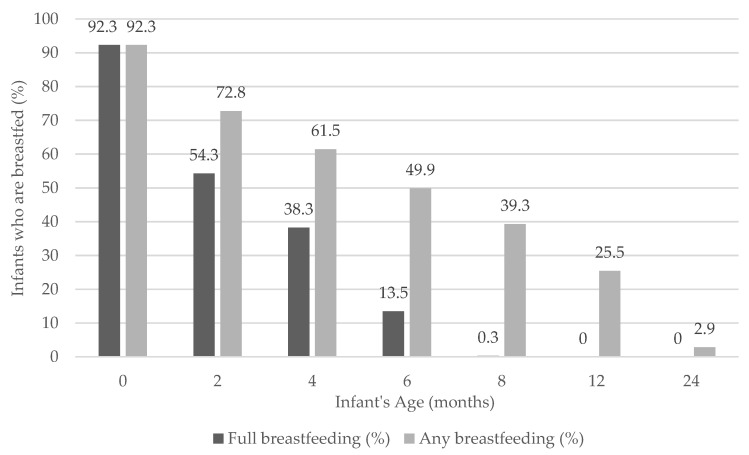
Prevalence of full and any breastfeeding by infant age.

Out of the 934 participants interviewed at two months, a predominant proportion of mothers (92.3%) had initiated breastfeeding. Figure 1 illustrates the prevalence of full and any breastfeeding by the age of the infants. At six months, 13.5% of the infants were fully breastfed, whereas almost half of the infants (49.9%) received some breast milk. Only 25.5% of the infants received some breast milk at 12 months, which dropped to 2.9% by 24 months. The median duration of full and any breastfeeding was 11 and 25 weeks, respectively.

In the univariable analysis, the duration of full breastfeeding at six months and any breastfeeding at 12 and 24 months was associated with potential family and infant characteristics (Supplementary Materials Table S1). Variables that were significant at *p* < 0.20 were maternal age, marital status of the mother, mother’s country of birth, maternal education, mother’s occupation, partner’s country of birth, socioeconomic status as per residential area in IRSAD, maternal employment status postpartum, method of child delivery, mother’s smoking status, and partner prefers breastfeeding.

In the multivariable analysis (Table 2), maternal education, socioeconomic status as per residential area in IRSAD, mother’s smoking status during pregnancy, and method of child delivery were significantly associated with the duration of both full breastfeeding at six months and any breastfeeding at 12 and 24 months. An increase in one year of mother’s age was associated with 2% decreased risk of stopping any breastfeeding at both 12 and 24 months (Adjusted Hazard Ratio (AHR) = 0.98). Compared to mothers who did not complete high school, those who had completed a university degree had a 35% lower risk of stopping full breastfeeding at six months (AHR = 0.65), and they had a 47% and 38% lower risk of ceasing any breastfeeding at 12 months (AHR = 0.53) and 24 months (AHR = 0.62) respectively. Mothers who resided in the least disadvantaged areas as per IRSAD had a 25% lower risk of discontinuing full breastfeeding at six months (AHR = 0.75), and they had a 26% and 23% lower risk of ceasing any breastfeeding at 12 months (AHR = 0.74) and 24 months (AHR = 0.77), respectively, compared to mothers who resided in the most disadvantaged areas. Mothers who were employed full-time at four months had a 68% higher risk of ceasing full breastfeeding at six months, compared to unemployed mothers (AHR = 1.68). Correspondingly, mothers who were employed full-time at 12 months had an 81% higher risk of ceasing any breastfeeding at 12 months (AHR = 1.81) and a 68% higher risk of stopping any breastfeeding at 24 months (AHR = 1.68), in comparison with unemployed mothers. Compared to mothers who did not smoke, those who smoked during pregnancy had a 51% higher risk of ceasing full breastfeeding at six months (AHR = 1.51), over two-fold increase in the risk of stopping any breastfeeding at 12 months (AHR = 2.11), and 94% higher risk of stopping any breastfeeding at 24 months (AHR = 1.94). Having a partner who preferred breastfeeding for the infant decreased the risk of discontinuing any breastfeeding by 21% at 12 months (AHR = 0.79) and 18% at 24 months (AHR = 0.82). Women who gave birth via a caesarean section had a 33% higher risk of stopping full breastfeeding at six months (AHR = 1.33), and they had a 31% and 22% higher risk of discontinuing any breastfeeding at 12 months (AHR = 1.31) and 24 months (AHR = 1.22), respectively, in comparison with women who had a vaginal delivery.

## 4. Discussion

This study provides valuable insight into the prevalence and determinants of breastfeeding duration for up to 24 months among mothers in South Western Sydney. The key predictors of breastfeeding duration were maternal age, maternal education, socioeconomic status, maternal employment status postpartum, mother’s smoking status during pregnancy, partner’s preference for breastfeeding, and method of child delivery. In this study, 92.3% of mothers had initiated breastfeeding, while only 49.9%, 25.5%, and 2.9% of all infants received some breast milk at 6, 12, and 24 months, respectively. The prevalence of breastfeeding at 6, 12, and 24 months is lower than the 60%, 42%, and 7% reported in the NIFS [12,29] and that reported for a cohort of South Australian children [30]. This could be attributed to a wide range of factors, including socioeconomic disadvantage faced by the women in this region.

In this study, only 13.5% of all infants were fully breastfed at six months, while 25.5% received some breast milk at 12 months and 2.9% received some breast milk at 24 months. These findings indicate that both the Australian [2,3] and WHO [1] breastfeeding recommendations are not being met in this community. Furthermore, the breastfeeding duration rates obtained through this study are consistent with previous studies conducted nationally and in other jurisdictions [13,24,31]. This indicates that, despite the recognised benefits of breastfeeding for up to 12 months and beyond, relatively few women in Australia achieve these breastfeeding targets. The Australian Government has undertaken various initiatives for the promotion of breastfeeding, including policy on promotion, protection, and support of breastfeeding, Baby-Friendly Hospital and Health Initiatives, Breastfeeding Support Clinics and Reference Groups, paid maternity leave policy, interventions for support of breastfeeding in the workplace, and support to vulnerable and at-risk mothers, among others [3]. Nevertheless, continuous monitoring of breastfeeding practices is essential to identify women who are at risk of stopping breastfeeding such that policymakers can act on the increasing health inequalities [13,24]. A recent study in Denmark has proposed an easy-to-use screening tool to predict mothers at risk of breastfeeding cessation within the first four months postpartum which may be useful for health professionals to identify at-risk mothers and hence provide additional support [32].

Maternal characteristics such as maternal age, education, socioeconomic status, and employment status have been shown to have a significant association with breastfeeding duration. This study identified factors associated with the risk of early cessation of breastfeeding among disadvantaged communities for whom breastfeeding promotion interventions can be targeted. Similar to the findings of other studies, an increase in years of maternal age reduced the likelihood of discontinuing any breastfeeding at both 12 and 24 months [13,21,30,31,33,34]. Previous studies suggest that older women may be in better circumstances, have higher education, be more financially secure, and may have prior breastfeeding experience, while younger mothers may be less knowledgeable about breastfeeding [35,36]. Consistent with other Australian and international studies [21,31,33,34,37,38], mothers with a higher educational level were more likely to continue breastfeeding. An increase in educational attainment may increase maternal knowledge on the infant health benefits of breastfeeding [36], which influences their intention to continue breastfeeding [39]. Moreover, an increase in health literacy levels, high income, higher level occupation, and provision of paid maternal leave may be some of the associated outcomes of a higher educational level, which may have an impact on infant feeding decisions [36].

Consistent with the findings of previous studies, women residing in areas of high socioeconomic disadvantage were more likely to cease breastfeeding [23,24,40]. The key reasons for lower breastfeeding duration rate among women with a low socioeconomic status may include low family support, reduced ability to obtain assistance for breastfeeding issues, rigid work schedule, and apprehensions of breastfeeding in public [24,41]. Similarly, mothers from a low socioeconomic background are more likely to socialise with women who are younger, less educated, and less inclined to breastfeed [24]. As a result, infants from lower-income families are more likely to fall ill and get hospitalised, and thus further increasing the health inequalities [24]. Especially in the context of South Western Sydney, ethnic minority groups and migrant population are more susceptible to these health inequalities as a result of low socioeconomic status and low levels of health literacy [18] and should be addressed while developing breastfeeding-promotion strategies.

In this study, mothers engaged in full-time employment were more likely to cease breastfeeding. Numerous studies have shown a negative association of maternal postnatal employment on breastfeeding duration [13,30,40,42,43,44]. The key barriers for employed mothers to continue breastfeeding include privacy issues, low support from their workplace, fatigue, lack of break time to express milk, inflexible work schedules, and inadequate facilities for pumping and storing milk [40,45,46]. Studies have suggested that breastfeeding-friendly workplaces increase the rate of breastfeeding duration among mothers who return to work postpartum [45,47]. Hence, health-promotion policies with effective translation around breastfeeding-friendly workplaces could significantly affect breastfeeding duration.

Consistent with earlier studies, women who smoked during pregnancy were more likely to discontinue breastfeeding early [13,21,48,49,50]. The negative impact of smoking on continuation of breastfeeding may be the result of suppression of prolactin levels by nicotine that reduces breast-milk supply [13]. However, as women who smoke are less likely to breastfeed, rather than a physiological effect, the negative association between smoking and breastfeeding duration could be attributed to psychosocial factors [51]. These social and behavioural factors among women who smoke include lower motivation to breastfeed, belief that smoking is a barrier to breastfeeding, and lower ability to seek help with breastfeeding difficulties [51]. Moreover, due to a clustering of unhealthy lifestyle practices among women from low socioeconomic groups, they may be less inclined to breastfeed [24]. Therefore, it is vital to educate pregnant mothers about the harmful effect of smoking on breastfeeding practices [52].

A partner’s preference for breastfeeding is recognised as an important factor that has a positive association with breastfeeding duration [13,30,33,34,53]. Practical and emotional encouragement from partners has been shown to be an essential component for successful breastfeeding, as they not only influence infant feeding decisions but increase the confidence of the mother [54]. Moreover, breastfeeding duration can be lengthened by establishing breastfeeding as a family practice and including partners in infant feeding decisions [55]. When partners share the mother’s difficulties in breastfeeding, infants have a better chance of receiving breast milk as per breastfeeding recommendations [54]. In this study, partners were not interviewed, and their breastfeeding preference was determined from the mother’s perspective only. Nevertheless, this finding emphasises the need to include partners in infant feeding decisions and breastfeeding-promotion interventions, to ensure prolonged breastfeeding duration.

Infant characteristics such as the method of child delivery had a significant association with breastfeeding duration. In this study, women who delivered via a caesarean section were more likely to discontinue full and any breastfeeding early, an outcome which is consistent with previous studies [33,35,53,56]. Various factors that result in early cessation of breastfeeding among mothers who deliver via a caesarean section include chronic pain, surgical trauma, anxiety, postpartum depression, and difficulties in correctly positioning the infant due to discomfort [57]. Any of these factors are likely to result in delayed initiation of breastfeeding and increased likelihood of an infant receiving formula in hospital, both of which have been associated with early cessation of full and any breastfeeding [56,58]. Hence, it is important to provide breastfeeding counselling and supplementary supportive care postpartum to women who deliver via a caesarean section [56].

This study has several strengths. First, this study had a good response rate of 69%, as a response rate of 50% is considered to be acceptable for validity of a study of this kind [59]. Furthermore, 90.24% of the mothers who consented to the study completed the interviews at two, four, and eight months, and 86.96% and 76.81% were retained in the study at 12 and 24 months, respectively. Second, this study provides valuable evidence on the prevalence and predictors of breastfeeding duration among women in South Western Sydney which was collected through a population-based sample from socioeconomically and ethnically diverse backgrounds. Third, this study used longitudinal data to identify the predictors of breastfeeding duration. Therefore, the findings of this study provide valuable insights into the population subgroups of women who do not continue to breastfeed, as well as potentially amendable risk factors of breastfeeding duration, which can be utilised to develop health promotion policies and strategies for prolonged breastfeeding duration, to ensure better health outcomes.

There are a few limitations of this study. First, as self-report was the basis for measurement of outcome, this may have given rise to a social-desirability bias. Second, mothers were not interviewed at around six months, but data were collected at four and eight months, and this may have led to some recall and measurement bias. However, maternal recall of breastfeeding duration has been shown to be valid and reliable, even after prolonged periods, such as three years [60]. Third, as the study sample consists of women who delivered in public hospitals located in South Western Sydney, the outcomes may not be generalisable to the entire population of South Western Sydney or Sydney. Fourth, factors related to the long-term health status of the mother, such as nutritional intake, existence of chronic diseases, and acute or chronic infections during the breastfeeding period, were not measured; thus, these factors may affect the study findings. Moreover, data on each participant’s education history about breastfeeding, such as attendance to antenatal classes, were not collected. Finally, the reasons for cessation of breastfeeding according to the infant’s age were not explored in-depth. Future studies should thoroughly investigate the primary reasons for discontinuing breastfeeding among the identified at-risk subgroups of women. This would aid in identifying the key barriers to breastfeeding and inform the development of interventions to ensure sustained breastfeeding duration. Nevertheless, this study endorses the necessity of increasing breastfeeding duration among women in Sydney.

## 5. Conclusions

This study reports the breastfeeding duration among women in South Western Sydney, one of Australia’s most culturally diverse and disadvantaged populations. Although breastfeeding initiation rates were high, the duration of breastfeeding was below the levels recommended in national and international guidelines. At six months, only about one in ten infants were fully breastfed, while almost half of the infants received some breast milk. Only about one quarter of the infants received some breast milk at 12 months, which dropped to around one in forty infants by 24 months. Lower maternal education level, lower socioeconomic status, full-time employment, maternal smoking during pregnancy, and caesarean delivery were associated with increased risk of stopping full breastfeeding at six months and any breastfeeding at 12 and 24 months. Older maternal age and partner’s preference for breastfeeding were associated with increased likelihood of continuing any breastfeeding at 12 and 24 months. This study highlights the critical determinants that influence recommended breastfeeding duration among mothers in South Western Sydney. Therefore, it is imperative that these factors are taken into account when developing health promotion policies and strategies to increase breastfeeding duration and ensure better health outcomes.

## Figures and Tables

**Table 1:** Family and infant characteristics of the participants with breastfeeding duration up to 24 months.

Characteristics	Total Participants (*n* = 934)	Full BF at 6 Months (*n* = 934)	Any BF at 12 Months (*n* = 900)	Any BF at 24 Months (*n* = 795)
	*n* (%)	*n* (%)	*n* (%)	*n* (%)
Family Characteristics *				
Marital status of mother				
Married	734 (78.6)	104 (14.2)	194 (26.4)	22 (2.9)
Living with a partner	110 (11.8)	18 (16.4)	29 (26.4)	4 (3.6)
Single	90 (9.6)	4 (4.5)	15 (16.7)	1 (1.1)
Mother’s country of birth				
Australia	437 (46.8)	63 (14.4)	109 (24.9)	12 (2.7)
China	57 (6.1)	6 (10.5)	16 (28.1)	0 (0.0)
Vietnam	133 (14.2)	13 (9.8)	25 (19.8)	4 (3.0)
Other Asian country	109 (11.7)	22 (20.1)	38 (34.9)	8 (7.3)
Middle East/Africa	81 (8.7)	2 (2.5)	17 (21.0)	1 (1.2)
Other	117 (12.5)	9 (7.7)	33 (2.8)	4 (1.7)
Maternal education				
Below year 12	168 (17.9)	10 (5.9)	16 (9.5)	3 (1.8)
Year 12 completed	192 (20.6)	13 (6.8)	36 (18.8)	6 (3.6)
College/TAFE	170 (18.2)	25 (14.7)	47 (27.6)	3 (1.8)
University	404 (43.3)	78 (19.3)	139 (34.4)	15 (3.7)
Mother’s occupation				
Home duties	169 (18.1)	18 (10.7)	33 (19.5)	5 (2.95)
Managerial	61 (6.5)	11 (18.0)	18 (29.5)	0 (0.0)
Professional	241 (25.8)	40 (16.6)	85 (35.3)	8 (3.3)
Sales/Clerical	295 (31.6)	39 (13.2)	66 (22.4)	10 (3.4)
Unskilled	168 (18.0)	18 (10.7)	36 (21.4)	4 (2.4)
Partner’s country of birth				
Australia	371 (39.7)	58 (15.6)	107 (28.8)	7 (1.9)
China	43 (4.6)	5 (11.6)	13 (30.2)	0 (0.0)
Vietnam	115 (12.3)	14 (12.1)	25 (21.7)	5 (4.3)
Other Asian country	106 (11.3)	19 (18.1)	33 (31.4)	8 (7.5)
Middle East/Africa	96 (10.3)	8 (8.3)	20 (20.8)	2 (2.1)
Other	121 (13.0)	18 (15.0)	27 (22.5)	4 (3.3)
Index for relative socioeconomic disadvantage ^a^				
Deciles 1 and 2	303 (32.4)	34 (11.2)	55 (18.2)	9 (3.0)
Deciles 3 and 4	220 (23.6)	24 (10.9)	57 (25.9)	3 (1.5)
Deciles 5 and 6	30 (3.2)	2 (6.7)	12 (40.0)	0 (0.0)
Deciles 7 and 8	160 (17.1)	24 (15.0)	49 (30.6)	6 (3.7)
Deciles 9 and 10	221 (23.7)	42 (19.0)	65 (29.4)	9 (4.1)
Parity				
Primiparous	465 (49.8)	77 (16.6)	120 (25.8)	15 (3.2)
Multiparous	469 (50.2)	49 (10.4)	118 (25.2)	12 (2.6)
Mother’s smoking status during pregnancy				
No	880 (94.2)	124 (14.1)	232 (26.4)	27 (0.8)
Yes	53 (5.7)	2 (3.8)	6 (11.3)	0 (0.0)
Mother’s alcohol consumption status during pregnancy				
No	832 (89.1)	111 (13.3)	209 (25.1)	23 (2.8)
Yes	100 (10.7)	15 (15.0)	29 (29.0)	4 (4.0)
**Infant Characteristics**				
Infant gender				
Male	477 (51.1)	58 (12.2)	121 (25.4)	8 (1.7)
Female	457 (48.9)	68 (14.9)	117 (25.6)	19 (4.2)
Infant birthweight				
>2500 g	887 (95.0)	119 (13.4)	226 (25.5)	25 (2.8)
<2500 g	47 (5.0)	7 (14.9)	12 (25.5)	2 (4.3)
Method of child delivery				
Vaginal	652 (69.8)	94 (14.4)	172 (26.4)	16 (2.4)
Caesarean section	281 (30.1)	32 (11.4)	62 (22.1)	11 (3.9)

* Maternal age is a continuous variable; therefore, the percentages have not been reported. ^a^ Decile 1 = most disadvantaged, and 10 = least disadvantaged. The total of the categories might not always add up to the total number of participants, due to missing or incomplete data for some items. *n:* sample size. BF: breastfeeding. TAFE: technical and further education.

**Table 2:** Factors independently associated with the risk of stopping full breastfeeding at six months and any breastfeeding at 12 and 24 months.

Variable **	Full BF at 6 Months (*n* = 934)			Any BF at 12 Months (*n* = 900)			Any BF at 24 Months (*n* = 795)		
	AHR	95% CI	*p*-Value	AHR	95% CI	*p*-Value	AHR	95% CI	*p*-Value
**Family characteristics**									
Maternal age (years)				0.98	0.96, 0.99	0.013	0.98	0.96, 0.99	0.003
Maternal education									
Below Year 12 *	1.00			1.00			1.00		
Year 12 completed	0.97	0.78, 1.20	0.754	0.96	0.72, 1.27	0.754	0.97	0.74, 1.26	0.799
College/TAFE	0.70	0.56, 0.88	0.002	0.68	0.51, 0.91	0.008	0.76	0.58, 0.98	0.037
University	0.65	0.53, 0.79	<0.001	0.53	0.41, 0.69	<0.001	0.62	0.49, 0.79	<0.001
Index for relative socioeconomic disadvantage ^a^									
Deciles 1 and 2 *	1.00			1.00			1.00		
Deciles 3 and 4	0.97	0.81, 1.16	0.757	0.75	0.60, 0.95	0.015	0.79	0.64, 0.97	0.023
Deciles 5 and 6	0.99	0.68, 1.45	0.956	0.58	0.35, 0.96	0.033	0.72	0.48, 1.09	0.120
Deciles 7 and 8	0.83	0.67, 1.02	0.071	0.62	0.48, 0.81	<0.001	0.70	0.53, 0.84	0.001
Deciles 9 and 10	0.75	0.62, 0.90	0.002	0.74	0.58, 0.94	0.015	0.77	0.62, 0.95	0.017
Maternal employment status at 4 months									
No *	1.00								
Casual employment	0.87	0.58, 1.31	0.516						
Part-time employment	1.72	1.26, 2.34	0.001						
Full-time employment	1.68	1.21, 2.35	0.002						
Maternal employment status at 12 months									
No *				1.00			1.00		
Casual employment				1.58	1.06, 2.36	0.026	1.13	0.77, 1.65	0.538
Part-time employment				1.48	1.21, 1.82	<0.001	1.42	1.87, 1.71	<0.001
Full-time employment				1.81	1.41, 2.32	<0.001	1.68	1.33, 2.11	<0.001
Mother’s smoking status during pregnancy									
No *	1.00			1.00			1.00		
Yes	1.51	1.13, 2.01	0.005	2.11	1.36, 3.26	0.001	1.94	1.29, 2.92	0.002
Partner prefers breastfeeding									
No *				1.00			1.00		
Yes				0.79	0.66, 0.95	0.010	0.82	0.70, 0.97	0.018
**Infant Characteristics**									
Method of child delivery									
Vaginal *	1.00			1.00			1.00		
Caesarean section	1.33	1.15, 1.53	<0.001	1.31	1.10, 1.57	0.003	1.22	1.03, 1.43	0.018

Variables that were significant at *p* < 0.2 and included in full multivariable model were maternal age, marital status of the mother, mother’s country of birth, maternal education, mother’s occupation, partner’s country of birth, socioeconomic status as per residential area in the Index of Relative Socioeconomic Advantage and Disadvantage (IRSAD), maternal employment status postpartum, method of child delivery, mother’s smoking status, and partner prefers breastfeeding. ** The final model consists of variables, which, when eliminated, cause a prominent deviance change (*p* < 0.05), as compared to the corresponding *X*^2^ test statistic on the relevant degrees of freedom. * Reference category. ^a^ Decile 1 = most disadvantaged and Decile 10 = least disadvantaged. BF: breastfeeding. AHR: Adjusted Hazard Ratio. 95% CI: 95% Confidence Interval.

## Supplementary Materials

The following are available online at https://www.mdpi.com/1660-4601/17/15/5384/s1. Table S1: Unadjusted association of family and infant characteristics with the risk of stopping full breastfeeding at six months and any breastfeeding at 12 and 24 months.
