# Supplementary material for: Determinants of Full Breastfeeding at 6 Months and Any Breastfeeding at 12 and 24 Months among Women in Sydney: Findings from the HSHK Birth Cohort Study
Source: Int J Environ Res Public Health. 2020 Jul 27;17(15):5384. doi: 10.3390/ijerph17155384 (PMC7432226; doi:10.3390/ijerph17155384)
Supplement: Supplementary file 1 [file ijerph-17-05384-s001.pdf]

Table S1. Unadjusted association of family and infant characteristics with the risk of stopping full breastfeeding at 6 months and any breastfeeding at 12 and 24 months.

Variable	Full BF at 6 Months (*n* = 934)			Any BF at 12 Months (*n* = 900)			Any BF at 24 Months (*n* = 795)		
	Crude HR	95% CI	*p*-Value	Crude HR	95% CI	*p*-Value	Crude HR	95% CI	*p*-Value
**Family Characteristics**									
Maternal age (years)	0.99	0.98, 1.01	0.230	0.97	0.96, 0.98	<0.001	0.97	0.96, 0.99	<0.001
Marital status of mother			<0.001			<0.001			0.002
Married	1.00			1.00			1.00		
Living with a partner	0.98	0.80, 1.20	0.852	1.04	0.83, 1.30	0.754	1.00	0.82,1.23	0.982
Single	1.65	1.32, 2.07	<0.001	1.56	1.23, 1.99	<0.001	1.53	1.23, 1.92	<0.001
Mother's country of birth			0.009			0.004			0.003
Australia	1.00			1.00			1.00		
China	0.99	0.75, 1.30	0.938	0.83	0.60, 1.14	0.245	0.93	0.71, 1.23	0.622
Vietnam	1.36	1.12, 1.65	0.002	1.23	0.99, 1.52	0.061	1.17	0.96, 1.42	0.127
Other Asian country	0.91	0.74, 1.13	0.398	0.72	0.56, 0.92	0.009	0.71	0.57, 0.88	0.002
Middle East/Africa	1.25	0.98, 1.59	0.069	1.19	0.92, 1.55	0.193	1.15	0.91, 1.47	0.237
Other	1.17	0.95, 1.44	0.131	0.92	0.73, 1.16	0.462	0.98	0.80, 1.20	0.837
Maternal education			<0.001			<0.001			<0.001
Below Year 12	1.00			1.00			1.00		
Year 12 completed	0.91	0.74, 1.12	0.363	0.79	0.63, 0.99	0.037	0.81	0.65, 0.99	0.044
College/TAFE	0.63	0.51, 0.78	<0.001	0.53	0.41, 0.67	<0.001	0.59	0.48, 0.74	<0.001
University	0.58	0.49, 0.70	<0.001	0.45	0.37, 0.55	<0.001	0.51	0.42, 0.61	<0.001
Mother's occupation			0.002			<0.001			0.001
Home duties	1.00			1.00			1.00		
Managerial	0.70	0.52, 0.94	0.018	0.69	0.50, 0.97	0.032	0.76	0.57, 1.02	0.707
Professional	0.70	0.57, 0.85	<0.001	0.60	0.48, 0.75	<0.001	0.68	0.56, 0.83	<0.001
Sales/Clerical	0.86	0.71, 1.04	0.129	0.86	0.70, 1.07	0.170	0.88	0.73, 1.07	0.198
Unskilled	0.90	0.73, 1.12	0.353	0.90	0.71, 1.14	0.388	0.96	0.77, 1.19	0.698
Partner's country of birth			0.010			0.039			0.045
Australia	1.00			1.00			1.00		
China	0.95	0.69, 1.30	0.747	0.86	0.60, 1.23	0.401	0.91	0.67, 1.25	0.581
Vietnam	1.37	1.11, 1.69	0.004	1.30	1.03, 1.64	0.031	1.14	0.92, 1.41	0.233
Other Asian country	1.03	0.83, 1.29	0.766	0.93	0.73, 1.20	0.591	0.79	0.63, 0.99	0.040
Middle East/Africa	1.38	1.10, 1.73	0.005	1.32	1.02, 1.69	0.029	1.20	0.96, 1.50	0.115
Other	1.16	0.95, 1.43	0.154	1.41	0.91, 1.44	0.264	1.07	0.87, 1.32	0.233
Index for relative socioeconomic disadvantage [a]			<0.001			<0.001			<0.001
Deciles 1 and 2	1.00			1.00			1.00		
Deciles 3 and 4	0.93	0.78, 1.11	0.416	0.79	0.65, 0.96	0.017	0.83	0.70, 1.00	0.044
Deciles 5 and 6	0.92	0.64, 1.35	0.681	0.59	0.37, 0.93	0.022	0.73	0.50, 1.06	0.103
Deciles 7 and 8	0.75	0.62, 0.91	0.004	0.62	0.50, 0.78	<0.001	0.70	0.58, 0.85	<0.001
Deciles 9 and 10	0.68	0.57, 0.81	<0.001	0.65	0.53, 0.79	<0.001	0.70	0.58, 0.83	<0.001
Intent to work or study 6 months postpartum			0.442			0.746			0.717
No	1.00			1.00			1.00		
Yes	1.06	0.91, 1.25	0.442	1.03	0.86, 1.23	0.746	0.97	0.83, 1.14	0.717
Maternal employment status at 4 months			0.021			0.266			0.045
No	1.00			1.00			1.00		
Casual employment	0.86	0.58, 1.28	0.454	0.82	0.52, 1.32	0.416	0.75	0.49, 1.14	0.182
Part-time employment	1.34	0.99, 1.82	0.058	1.08	0.77, 1.52	0.655	1.03	0.76, 1.40	0.831
Full time employment	1.50	1.08, 2.08	0.017	1.38	0.96, 1.97	0.083	1.28	0.92, 1.79	0.141
Maternal employment status at 12 months			0.008			0.081			0.098

No	1.00			1.00			1.00		
Casual employment	1.06	0.75, 1.50	0.745	1.21	0.83, 1.77	0.327	0.89	0.62, 1.27	0.508
Part-time employment	0.92	0.79, 1.08	0.312	1.10	0.92, 1.32	0.315	1.10	0.93, 1.29	0.268
Full time employment	1.36	1.11, 1.66	0.004	1.33	1.06, 1.66	0.013	1.270	1.03, 1.56	0.023
Parity			0.003			0.241			0.208
Primiparous	1.00			1.00			1.00		
Multiparous	1.21	1.07, 1.38	0.003	1.09	0.94, 1.26	0.241	1.09	0.95, 1.24	0.208
Mother's smoking status during pregnancy			<0.001			<0.001			<0.001
No	1.00			1.00			1.00		
Yes	1.69	1.28, 2.23	<0.001	2.02	1.50, 2.71	<0.001	1.88	1.42, 2.48	<0.001
Mother's alcohol consumption status during pregnancy			0.193			0.398			0.467
No	1.00			1.00			1.00		
Yes	0.87	0.71, 1.07	0.194	0.90	0.71, 1.15	0.398	0.92	0.77, 1.14	0.467
Infant feeding decision made before pregnancy			0.530			0.970			0.856
No	1.00			1.00			1.00		
Yes	1.06	0.89, 1.27	0.530	1.00	0.82, 1.23	0.970	0.98	0.82, 1.18	0.856
Partner prefers breastfeeding			0.017			0.005			0.045
No	1.00			1.00			1.00		
Yes	0.83	0.72, 0.97	0.017	0.79	0.67, 0.93	0.005	0.86	0.73, 1.00	0.045
**Infant Characteristics**									
Infant gender			0.527			0.848			0.467
Male	1.00			1.00			1.00		
Female	0.96	0.84, 1.09	0.527	0.99	0.85, 1.14	0.848	0.953	0.84, 1.09	0.467
Infant birthweight			0.379			0.868			0.973
>2500g	1.00			1.00			1.00		
<2500g	1.14	0.85, 1.54	0.379	1.03	0.73, 1.45	0.868	1.00	0.74, 1.36	0.973
Method of child delivery			0.003			0.008			0.070
Vaginal	1.00			1.00			1.00		
Caesarean section	1.24	1.08, 1.43	0.003	1.24	1.06, 1.45	0.008	1.14	0.99, 1.31	0.070
Gestational Age			0.163			0.408			0.353
Premature (<37 weeks)	1.00			1.00			1.00		
Normal (>37 weeks)	0.84	0.66, 1.07	0.163	0.89	0.68, 1.17	0.408	0.89	0.69, 1.14	0.353

BF: Breastfeeding. HR: Hazard Ratio. 95% CI: 95% Confidence Interval. [a] 1 = most disadvantaged and 10 = least disadvantaged.
